# Does bisphenol A (BPA) participates in the pathogenesis of Polycystic Ovary Syndrome (PCOS)?

**DOI:** 10.1016/j.clinsp.2023.100310

**Published:** 2023-11-25

**Authors:** Lorena Ana Mercedes Lara Urbanetz, José Maria Soares Junior, Gustavo Arantes Rosa Maciel, Ricardo dos Santos Simões, Maria Cândida Pinheiro Baracat, Edmund Chada Baracat

**Affiliations:** Gynecology Division, Hospital das Clínicas, Faculdade de Medicina, Universidade de São Paulo (HCFMUSP), São Paulo, SP, Brazil

**Keywords:** Bisphenol A, Endocrine disruptors, Hyperandrogenism, Insulin resistance, Obesity, Polycystic Ovary Syndrome

## Abstract

PCOS is an endocrine disorder characterized by chronic anovulation, hyperandrogenism, and polycystic ovaries. Its etiology is uncertain. It is debated whether BPA would be a component of the environmental factor in the etiology of PCOS. Contamination by BPA can occur from food packaging (exposure during the diet) and through skin absorption and/or inhalation. It can be transferred to the fetus via the placenta or to the infant via breast milk, and it can be found in follicular fluid, fetal serum, and amniotic fluid. The phenolic structure of BPA allows it to interact with Estrogen Receptors (ERs) through genomic signaling, in which BPA binds to nuclear ERα or Erβ, or through nongenomic signaling by binding to membrane ERs, prompting a rapid and intense response. With daily and constant exposure, BPA's tendency to bioaccumulate and its ability to activate nongenomic signaling pathways can alter women's metabolic and reproductive function, leading to hyperandrogenism, insulin resistance, obesity, atherogenic dyslipidemia, chronic inflammatory state, and anovulation and favoring PCOS. The harmful changes caused by BPA can be passed on to future generations without the need for additional exposure because of epigenetic modifications. Not only high BPA levels can produce harmful effects, but at low levels, BPA may be harmful when exposure occurs during the most vulnerable periods, such as the fetal and neonatal periods, as well as during the prepubertal age causing an early accumulation of BPA in the body. Learning how BPA participates in the pathogenesis of PCOS poses a challenge and further studies should be conducted.

## Highlights

• Bisphenola A (BPA) is an endocrine disruptor widely investigated as a possible environmental contributor to the pathogenesis of Polycystic Ovary Syndrome (PCOS).
• Due to its structural similarity with the Estrogen molecule (E2), it acts as a xenoestrogen, binding to genomic and non-genomic estrogen receptors, causing metabolic and hormonal changes that lead to PCOS (hyperandrogenism, insulin resistance, hyperinsulinemia, obesity, atherogenic dyslipidemia, anovulation, and ovarian cysts).
• The aim and of this review were to present the mechanisms of bisphenol A participation in the pathogenesis of Polycystic Ovary Syndrome.

## Endocrine-disrupting chemicals

An endocrine disruptor was defined as: “an exogenous chemical, or a mixture of chemicals, that interferes with any aspect of hormone action”, consequently causing adverse health effects.^1^

The Endocrine-Disrupting Chemicals (EDCs) can affect not only the exposed individuals but also their children and subsequent generations. In the United States, more than 6 million pounds of Bisphenol A (BPA) are produced every year and it is considered the third most important environmental contaminant according to the US Environmental Protection Agency (EPA).^2^

BPA may be associated with Polycystic Ovary Syndrome (PCOS), infertility in women, altering the morphology and function of the oviduct, uterus, ovary, and hypothalamic-pituitary-ovarian axis in animal models. Additionally, BPA can disrupt embryo implantation.^3^

Understanding how BPA, the most widespread endocrine disruptor in nature, participates in the pathogenesis of PCOS, is the aim of this review.

## Polycystic Ovarian Syndrome (PCOS)

PCOS is an endocrine alteration of uncertain etiology. A few of the etiologic hypotheses for the development of PCOS are hormonal imbalance, epigenetic and genetic defects, and environmental factors. It is debated whether BPA would be a component of the environmental factor in the etiology of PCOS. Exposure during the prenatal period to elevated levels of Anti-Müllerian Hormone (AMH), androgens, and endocrine disruptors such as BPA may also participate in the etiopathogenesis of PCOS.^4^

PCOS is clinically characterized by hormonal alterations (chronic anovulation, hyperandrogenism, and polycystic ovarian morphology) and metabolic alterations characteristically referred to as obesity, Insulin Resistance (IR), atherogenic dyslipidemia, and metabolic syndrome.^5^ PCOS occurs in 6% to 20% of reproductive-age women.^4^

Women with PCOS are at an increased risk of pregnancy complications and adverse outcomes in the offspring that could be related to factors involved in the pathogenesis of the Syndrome and the related comorbidities.^6^ The hyperandrogenism present in PCOS can affect reproductive functions, not only ovarian folliculogenesis but also endometrial receptivity and the establishment and maintenance of pregnancy.^7^

The Rotterdam Consensus published by the American Society for Reproductive Medicine (ASRM), along with the European Society of Human Reproduction and Embryology (ESHRE), in 2004 defined that a diagnosis of PCOS requires at least two of the following three criteria: oligoovulation and/or anovulation, clinical and/or biochemical evidence of hyperandrogenism, and morphology of polycystic ovaries. To confirm the syndrome, other causes of chronic anovulation should be ruled out, and so should the disorders that mimic the clinical features of PCOS, such as thyroid disorders, hyperprolactinemia, nonclassical congenital adrenal hyperplasia, androgen-secreting tumors, and Cushing's syndrome.^8^

## Bisphenol A (BPA)

Bisphenol A is considered an estrogen-like endocrine-disrupting chemical. It was first developed as a synthetic estrogen in the 1890s. It was later used as a monomer in the production of polycarbonate plastics and as an intermediate in the synthesis of epoxy. Polycarbonate is used in the composition of various objects such as CDs, cups, plastic bottles, baby bottles, etc., while epoxy resins are mainly used to coat the inside of food cans and in dental fillings. BPA is also used as a developer in thermal papers.^9^

Some studies have reported that BPA exposure is associated with reproductive disorders, including obstetric complications such as those seen in women with PCOS.^10^ BPA would be an ovarian toxicant that decreases oocyte quality in animal models and women undergoing In Vitro Fertilization (IVF), as well as a uterine toxicant because it decreases uterine receptivity and increases implantation failure in animal models. Exposure to BPA may be associated with adverse birth outcomes, hyperandrogenism, sexual dysfunction, and impaired implantation in humans.^11^

Daily, high-level exposure to BPA occurs mainly through the diet when the chemical leaches into foods and beverages from their packaging. Exposure to BPA also occurs through skin absorption and inhalation and it may be found in follicular fluid, fetal serum, and amniotic fluid. Transmission of BPA from the pregnant woman to the fetus during pregnancy through the placenta and to the child through breast milk has been demonstrated.^12^ These findings suggest early BPA accumulation and significant exposure.

BPA has a short half-life of 6 hours and is inactivated through glucuronidation by uridine diphosphate glucuronosyl-transferases and sulfation, by phenol-sulfotransferases in hepatocytes microsomes and it is eliminated as BPA-glucuronide (inactive form), primarily in the urine. However, BPA levels do not drop as quickly as expected after fasting, which suggests that BPA enters the human body through non-dietary routes or is considered persistent due to widespread and continuous population exposure.^13^

Periods of human development, including the prenatal period and infancy, are critical in terms of sensitivity to the effects of BPA. This is because fetuses and babies, unlike adults, do not have protective mechanisms such as DNA repair capacity, a fully functional blood-brain barrier, hepatic metabolism, and a competent immune system.^14^ The increased sensitivity to BPA during fetal and neonatal development can be explained by the liver's limited capacity to conjugate (deactivate) BPA.

In tissues such as the lungs, liver, kidneys, and placenta of animals and humans, the β-glucuronidase enzyme cleaves the glucuronide group and ensures the deconjugation of BPA returning to its active form that circulates again through the body. During pregnancy, the conjugated form of BPA crosses the placenta and undergoes deconjugation and the fetus is exposed to the active form of BPA.^15^

The tolerable BPA daily intake proposed by the US Environmental Protection Agency (50 µg/kg/day) and the European Food Safety Authority (4 µg/kg/day) were based on the Lowest Observable Adverse Effect Level (LOAEL). Nevertheless, plentiful scientific evidence has accumulated to show that BPA can interfere with the endocrine signaling pathways at doses lower than the calculated safe dose.^16^ The harmful effects caused by low-dose BPA in fetuses and newborns can be transmitted to third or fourth generations. The suggested mechanism of transgeneration is epigenetic changes.^17^

## Bisphenol A and its action on estrogen receptors

BPA is an exogenous chemical substance that mimics the activity of 17-β estradiol and it is considered a xenoestrogen. Consequently, BPA is able to disrupt E2 feedback at the hypothalamus-pituitary level and also at the ovarian level, thereby suppressing HPO axis functions.^18^ BPA can also cause inadequate hormone production, menstrual cycle abnormalities, infertility, and impairing the development and functioning of the reproductive system.^19^

The affinity of BPA for nuclear Etrogen Receptors (ERs) appears to be lower than 17-beta Estradiol (E2), but its estrogen potency equals that of E2 in the nonnuclear ER-mediated responses which are intense and rapid even at very low concentrations.^20^ BPA may trigger estrogen-activated pathways by binding to membrane ERs (nonnuclear ERs), such as transmembrane ERs like GPR30 (G Protein-coupled Receptor 30).^18^

Kawa et al.^14^ related that BPA also would have the ability to interact with Estrogen-Related Receptors gamma (ERRg) which are nuclear receptors expressed highly in the placenta, fetus, and neonate; with Aryl Hydrocarbon Receptor (AhR) that are able to mediate the toxicity of various EDCs; androgen receptors causing an anti-androgenic effects and thyroid receptors with agonistic and antagonistic effects). BPA may have an anti-estrogenic effect by inhibiting the activity of the aromatase.^21^

## Bisphenol A and changes in steroidogenesis/hyperandrogenism

BPA appears to alter the gene expression of some PCOS-related genes and downregulates the level of their m-RNA transcribed altering the Hypothalamic-Pituitary-Ovarian axis (HPO axis), steroidogenic and metabolic pathways.^22^

The dysregulation of gene expression caused by BPA exposure, at the hypothalamus and pituitary level impacts the functioning of the ovary.^22^ The chemical's activation of the Gonadotropin-Releasing Hormone (GnRH) pulse generator is thus exaggerated, which induces a constant increase in LH and a decline in FSH secretion through the hypophysis, harming follicle development and increasing ovarian androgen production. BPA also could alter the expression of genes of ovarian steroidogenesis increasing RNAm expression of key enzymes such as 17α-hydroxylase, leading to hyperandrogenism and ovulatory dysfunction.^23^

The BPA can directly prompt androgen production in the ovarian theca cells, causing hyperandrogenism, and it can also interact with receptors in adipose tissue and stimulate pancreatic beta cells to produce insulin, leading to hyperinsulinemia, which results in the accumulation of lipids in adipose tissue. All these effects harm ovarian folliculogenesis, bringing about anovulation and favoring PCOS.^23^

It has also been shown that BPA can have antiestrogen action by inhibiting the activity of aromatase. Such an inhibition heightens testosterone levels and causes hyperandrogenism.^21^

BPA may also interact with SHBG and is also capable of displacing sex steroids from SHBG thus increasing the quantity of free testosterone. Rutkowska et al.^24^ believe that increased serum concentration of BPA in women with PCOS compared to healthy controls, reduced levels of SHBG in the PCOS group, it is already known that it would be the action of insulin and androgens.

BPA exposure was negatively associated with peak serum estradiol levels during gonadotropin stimulation, number of recovered oocytes, number of normally fertilized oocytes, and implantation.^25^ BPA has been linked to a decreased antral follicle count in infertile women with PCOS suggesting a role for BPA in impairing the ovarian reserve.^26^ When exposed to high doses of BPA, changes in follicular growth may occur, leading to enlarged and atretic follicles with a decrease in the number of antral follicles.^27^

## Bisphenol A and chronic inflammatory state

Adipose tissue is a fat storage reservoir, but it is also an endocrine organ that secretes adipokines, cytokines, and chemokines. Bisphenol A likely acts through ERs on the adipocytes and macrophages that infiltrate the adipose tissue, promoting a chronic inflammatory state.^22^

It has been found that BPA is capable of inhibiting adiponectin secretion in adipose tissue as well as stimulating Interleukin 6 (IL-6) and Tumor Necrosis Factor α (TNF-α) secretion. Inflammation and oxidative stress may further aggravate carbohydrate metabolism disorder including disrupted insulin signaling in adipose tissue and glucose intolerance.^28^

In the study by Ariemma et al.,^29^ 3T3-L1 preadipocytes were exposed to 1 nM of BPA for two weeks before adipogenesis induction and found an increase in fat accumulation following stimulation of adipogenesis and reduced insulin-stimulated glucose uptake with an increase in mRNA levels of inflammatory markers such as IL6, INFγ.

Bisphenol A can stimulate cytokine production and the proliferative response of spleen and thymus cells in vitro. Besides, it is involved in the production of autoantibodies by β1 cells and may cause a rise in the incidence of autoimmune diseases. Dong et al.^30^ related that BPA exposure increased the concentration of serum anti-dsDNA antibody and IL-17, and the level of RORγt protein (the transcription factor of Th17 cells) in MRL/lpr mice (Lupus-prone mice) and it may induce the development of Systemic Lupus Erythematosus (SLE). In SLE, inflammation, autoantibodies, and estrogen are important pathological mechanisms.

BPA acting as a xenoestrogen could alter the subset of T-cells, B-cell function, and dendritic cell activity, inducing abnormal immune response with alteration of the transcription of the target gene and disruption of ER and PPAR signaling.^31^

Tarantino et al.^32^ found that, regardless of age and Body Mass Index (BMI), higher serum BPA levels were detected in a subgroup of women with PCOS who had more severe IR and hyperandrogenism, a higher prevalence of hepatic steatosis, and evidence of a chronic low-grade inflammatory state.

## Bisphenol A and insulin resistance

Estrogen receptors ER-α and ER-β are present in the islet of Langerhans and E2 modulate insulin secretion. It appears that BPA can regulate insulin concentration in the pancreas by activating ER-α with a similar response to E2. ER-α would be the main ER involved in the regulation of insulin secretion by E2 and BPA. Increased insulin gene expression and insulin release in response to stimulatory glucose concentrations, appears to be one of the mechanisms responsible for this estrogenic effect of BPA.^20^

The endocrine pancreas is the target of BPA exposure with possibly different mechanisms depending on whether exposure occurs during fetal life or adulthood. Indeed, fetal pancreas differentiation appears to be highly sensitive to BPA exposure based on researched results, e.g., β-cell proliferation and apoptosis.^33^

BPA appears to alter insulin synthesis and/or release by pancreatic β-cells and insulin signaling within insulin-sensitive organs (liver, muscle, adipose tissue). This resulted in variations in the expression of liver or adipose tissue-specific markers, which are indicative of an IR state. IR leads to an increase in lipolysis with a consequent increase in the plasma concentration of free fatty acids, an increase in hepatic glucose production, and a decrease in muscle glucose uptake with an increase in concentration. of blood glucose.^33^

Exposure to BPA appears to decrease the sensitivity of adipocytes to insulin due to a decrease in Glucose Transporter 1 (GLUT1) expression and insulin receptor phosphorylation, suggesting that BPA may alter glucose metabolism and favor the onset of DM2.^34^

In Skeletal muscle and in hepatocytes, the Insulin Receptor Substrate 1 (IRS1) is upregulated by exposure to BPA under basal conditions.^35^ BPA also reduces glucokinase activity while estrogen stimulates its activity under the same conditions.^36^ In this way, exposure to BPA can alter insulin signaling pathways.

Martinez-Pinna et al.^37^ showed that β-murine cells exposed to BPA presented an imbalance in their electrical activity with a decrease in Na+ and K+ currents due to the deregulation of several genes that codify components of the Na+ and K+ channels. This change in the electrical activity of β cells could impair insulin secretion with diabetogenic effects.

Inflammation and oxidative stress caused by BPA exposure may also favor disruptive effects on glucose metabolism leading to glucose intolerance by disruption of insulin signaling in adipose tissue.^28^

## Bisphenol A and obesity

An excessive intake of energy and a sedentary lifestyle are known to be risk factors for gaining weight; nevertheless, there has been a growing interest in the effects environmental chemicals may have on the development of obesity. Given that the adipose tissue is considered an endocrine organ, it may be targeted by EDCs. When EDCs stimulate adipogenesis and obesity are termed “obesogens”.^38^ Some EDCs may exert obesogenic effects by altering energy homeostasis.^39^

A particularly sensitive period of exposure is the intrauterine or neonatal period; early exposure to BPA is associated with child obesity.^40^ Chronic intrauterine exposure to low BPA dose may not only trigger obesity but also contribute to IR and chronic low-grade inflammation in women who will develop PCOS. A correlation between BMI and BPA exposure has been reported.^41^

Obesogens favor adipogenesis through such mechanisms as follows: an increase in the number and size of adipocytes; altering basal metabolism and hormones that regulate appetite and satiety; favoring calorie storage and altering insulin sensitivity in the liver, skeletal muscle, brain, pancreas, and gastrointestinal system.^39^^,^^42^

BPA can also bind to nuclear receptors like the Peroxisome Proliferator-Activated Receptors (PPARs), favoring obesity by altering gene expression. The PPARs regulate adipocyte proliferation and/or differentiation.^39^

Prenatal exposure to BPA in animals enhances serum leptin levels and decreases serum adiponectin levels contributing to adipocyte disfunction. These results may explain lipid accumulation and the increase in fat mass and obesity.^43^

BPA suppressed adiponectin release as efficiently or even more than E2 at equimolar concentrations. Although transcriptional stimulation of the Glucocorticoid Receptor (GR) is considered essential for adipocyte differentiation, BPA was shown to induce the differentiation of human adipocytes through a nonclassical ER pathway in the absence of exogenous glucocorticoids.^44^

BPA as an obesogenic not only alters BMI, it also affects adipose tissue and glucose and lipid metabolism.^45^

## Bisphenol A and dyslipidemia

Exposure to BPA in women with PCOS increases the metabolic risk causing obesity, mainly visceral, insulin resistance, hyperinsulinemia, dyslipidemia, and hyperandrogenism.^46^

Animal studies suggest that BPA has the potential to induce lipid disorders. For example, early exposure to BPA showed an increase in the circulating levels of Total Cholesterol (TC), Triglycerides (TG), and LDL-C and a reduction in the HDL-C levels, characterizing atherogenic dyslipidemia.^47^

In a 5-year prospective study, Li et al. (2020) measured the serum concentrations of BPA, TGs, LDL-C, and HDL-C and found that greater exposure to BPA is associated with a higher prevalence of low HDL cholesterol.^48^

## Bisphenol A and epigenetic changes

BPA may exert them through epigenetic mechanisms which include DNA methylation, histone modifications, and changes in micro RNA expression.^14^

Epigenetic modification can only occur if exposure to BPA occurs during the early developmental stage (critical developmental window), predisposing the subsequent generations to transgenerational inheritance of the diseases.^14^ BPA's deleterious effects vary depending on doses, route of administration, exposure period and animal models.^25^

Puttabyatappa et al.^49^ observed in female sheep offspring, that prenatal treatment with BPA deregulated the expression of 194 genes in the liver and 112 genes in muscle as well as 155 mitochondria-related genetic pathways in both liver and muscle; 1415 genetic pathways that participate in oxidative stress and lipid biosynthetic process in the liver; 192 genetic pathways related to RNA biosynthetic processes in muscle. These findings may explain the development and/or maintenance of defects contributing to BPA-induced prenatal metabolic dysfunctions.^49^

## Conclusions

Bisphenol A is considered a xenoestrogen that can bind to nuclear ERs (genomic signaling) as well as to membrane ERs (nongenomic signaling), where it elicits a rapid and intense response.

As exposure to BPA is chronic (daily and constant) with a tendency toward bioaccumulation and given that BPA can activate nongenomic signaling pathways quickly and intensely, BPA levels from environmental exposure may provoke harmful effects, such as hyperandrogenism, IR, obesity, atherogenic dyslipidemia, chronic inflammatory state, and anovulation, contributing to PCOS. Changes induced by BPA may be passed on to future generations without the need for additional exposure because of it's transgenerational effects and epigenetic modifications. Not only high BPA levels can produce harmful effects, but at low levels, BPA may be harmful when exposure occurs during the most vulnerable periods, such as the fetal and neonatal periods, as well as during the prepubertal age, causing an early accumulation of BPA in the body. Understanding how BPA participates in PCOS pathogenesis poses a challenge and further studies should be conducted.

## Declaration of Competing Interest

The authors declare no conflicts of interest.
